# Impact of Lipid Bilayer Composition and Physicochemical Properties on Constitution of a Transmembrane Helical Peptide into Exosome-Mimetic Vesicles

**DOI:** 10.1021/acs.molpharmaceut.5c00825

**Published:** 2025-10-08

**Authors:** Shiho Tsutsumi, Yuki Takechi-Haraya, Yasuhiro Abe, Kohsaku Kawakami

**Affiliations:** † Research Center for Macromolecules and Biomaterials, 52747 National Institute for Materials Science, 1-1 Namiki, Tsukuba, Ibaraki 305-0044, Japan; ‡ Graduate School of Science and Technology, University of Tsukuba, 1-1-1 Tennodai, Tsukuba, Ibaraki 305-8577, Japan; § Analytical Research Laboratory, Eisai Co. Ltd., 5-1-3 Tokodai, Tsukuba, Ibaraki 300-2635, Japan; ∥ Division of Biochemistry, National Institute of Health Sciences, 3-25-26 Tonomachi, Kawasaki, Kanagawa 210-9501, Japan; ⊥ Division of Drugs, National Institute of Health Sciences, 3-25-26 Tonomachi, Kawasaki, Kanagawa 210-9501, Japan

**Keywords:** exosomes, exosome-mimetic vesicles, lipid membrane, helical peptide

## Abstract

Exosomes are expected to efficiently deliver drugs, such as microRNAs and proteins, to targeted organs. However, using natural exosomes presents many difficulties in terms of safety, quality control, and manufacturing; therefore, developing exosome-mimetic artificial materials is desirable. In this study, we elucidated how sphingomyelin (SM) and cholesterol (CH), the main constituents of the exosome membrane, in addition to phosphatidylcholine (PC), influence the physicochemical properties of PC vesicles. Then, the relevance of these properties to the secondary structure and insertion efficiency of a helical peptide, the transmembrane domain of integrin α, was investigated. The constitution of this peptide was most successful with exosome-mimetic vesicles (EMV) bearing 15 mol % SM and 40 mol % CH, and the exclusion of SM or CH resulted in low dispersion stability or unsuccessful peptide constitution. Physicochemical analysis of the membrane properties revealed that successful peptide incorporation into the lipid membrane relied on the membrane softness induced by CH and the appearance of a highly mobile boundary phase induced by SM, which together created a favorable environment for the peptide. These results provide important insights that serve as a foundation for developing EMV as drug carriers.

## Graphical abstract

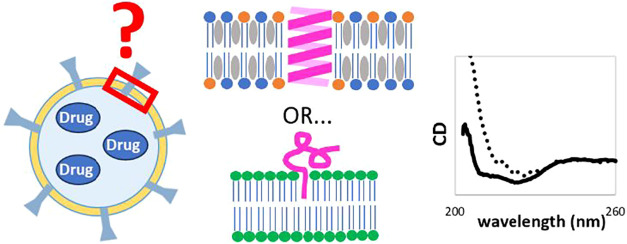

## Introduction

As our understanding of exosome functions has deepened, research on exosome-based drug delivery has grown.^1^ Exosomes are cell-secreted, small vesicles of approximately 50–150 nm in diameter and are known to deliver signaling molecules, such as microRNAs and proteins, to distant target organs.^2^ This function makes exosomes attractive as a drug delivery vehicle. The molecular mechanism of their organotropism has been partially revealed. The combination of integrin α and β subunits is involved in the determination of target organs;^3^ at least 18 α and eight β subunits are known in the human integrin family, whose principal structural features are well conserved.^4^

However, the use of exosomes as pharmaceutical products presents some problems. First, only autologous exosomes are feasible for administration due to safety concerns.^1^ The manufacturing cost is high even if sufficient amounts of drug-encapsulated donor-derived exosomes are produced. Second, quality control of exosomes is challenging, as their heterogeneity and functions are not yet well-understood.^2^ Thus, fully designed exosome-mimetic vesicles (EMV) with the desired functions are preferable.

Preferential accumulation of fully designed EMV decorated with Integrin α6β4 in the lung of mice has been reported, together with the successful delivery of encapsulated microRNAs to the recipient cells.^5^ Lipids used for this EMV were egg phosphatidylcholine (PC), egg sphingomyelin (SM), cholesterol (CH), and C_16_ ceramide. This study validated not only the function of integrin but also the design and concept of exosome-mimetic vesicles. Another report showed higher cellular uptake of EMV than PC/CH liposomes by the A549 cell line and successful gene silencing by encapsulated small-interfering RNA (siRNA).^6^ This EMV was formulated with a mixture of unsaturated phospholipids and CH without any targeting ligand. This report highlights the advantages of exosome-like lipid compositions over simple PC/CH formulations.

To develop EMV as a pharmaceutical product, its physicochemical characteristics must be understood and controlled. For example, thermodynamic properties, membrane polarity, phase separation, particle size, ζ potential, and the ability to retain functional proteins on the membrane likely influence EMV pharmacokinetics and storage capability. An important consideration here is that the physicochemical properties of the membrane are likely to influence both the secondary structure of the integrin transmembrane domain and its structural stability within the membrane, as well as the loading efficiency of integrins. However, physicochemical studies on exosome membranes or exosome-mimetic membranes are limited, and it remains unclear whether the membrane properties affect the secondary structure and stability of the integrin transmembrane domain, despite numerous reports on the reconstitution of integrins into lipid membranes and despite numerous studies on the lipid raft. The lipid raft is a membrane-protein domain on the cell membrane, enriched in CH and high-melting temperature (*T*_m_) lipids such as sphingolipids,^7^ forming the liquid-ordered (l_o_) phase that is hard to solubilize.^8^ The lipid raft is phase-separated from the liquid-disordered (l_d_) phase consisting of low-*T*_m_ lipids,^9–12^ and it is widely recognized that some membrane proteins localize at the lipid raft. Therefore, the addition of both the sphingolipid and the CH might be important for the stable reconstitution of integrin to the EMV. From a practical point of view, the PC species used in marketed drug formulations are typically saturated lipids such as dipalmitoylphosphatidylcholine (DPPC) or distearoylphosphatidylcholine (DSPC), because the pharmaceutical products generally require long-term stability over several years. The optimal design of EMV would not be an accurate mimic of exosomes using biorelevant lipids, which are typically unsaturated lipids, but would include the acquirement of vesicles with similar functions using these pharmaceutically relevant PC species.

Previous studies of EMV did not confirm whether the transmembrane domain of the reconstituted integrin forms its expected α-helical structure stably within the membrane. This is likely because it is technically difficult to selectively analyze the structure of the transmembrane domain within the full-length protein. Also, the physicochemical evaluations were limited in the previous studies on EMVs, as they generally focused more on efficacy and biodistribution. In this study, we investigated the effects of SM and CH on the physicochemical properties of the PC membrane. This combination of lipids was selected based on a lipidomic study of exosomes, which reported a larger proportion of SM (approximately 15 mol %) and CH (approximately 40 mol %) in the exosome membrane than in the cell membrane of its origin.^13^ For PC, DPPC was used in this study because it is chemically stable and its acyl chain length is identical to most of the egg SM. Use of an unsaturated lipid such as 1-palmitoyl-2-oleoyl-*sn*T-glycero-3-phosphocholine (POPC) was not considered, as it is not used in approved liposomal formulations due to their oxidative instability. Phosphatidylserine is also an important exosomal component. However, this study did not consider its inclusion in EMV, as it accelerates clearance of the vesicle from blood by promoting macrophage phagocytosis.^14^ Then, we investigated the impact of the membrane properties on the constitution of a peptide corresponding to the transmembrane domain of integrin α using circular dichroism (CD) spectroscopy and ζ potential measurements in order to find the optimal design of exosome-mimetic vesicles.

## Methods

### Materials

DPPC and CH were purchased from Nippon Oil and Fat (Tokyo, Japan) and Sigma-Aldrich (St. Louis, MO, USA), respectively. Egg SM and dipalmitoylphosphatidylserine (DPPS) were obtained from Avanti Polar Lipids (Alabaster, AL, USA). 1-[6-(Dimethylamino)­naphthalen-2-yl]­dodecan-1-one (laurdan) and sucrose were supplied by Cayman Chemical (Ann Arbor, MI, USA) and FUJIFILM Wako Pure Chemical (Osaka, Japan), respectively. 3-[(3-Cholamidopropyl)­dimethylammonio]-1-propanesulfonate (CHAPS) was purchased from Nacalai Tesque (Kyoto, Japan). A peptide with an amino acid sequence of the transmembrane domain of integrin α (ERAIPIWWVLVGVLGGLLLLTILVLAMWKVGFFKRNRPP) was chemically synthesized by Hokkaido System Science (Sapporo, Japan). All reagents used in this study were of reagent grade and used as supplied.

The molecular weight of the synthesized peptide was confirmed using a TripleTOF 6600 plus time-of-flight mass spectrometer (Sciex, Framingham, MA, USA) by direct infusion of the peptide solubilized in a methanol–chloroform mixture. Monoisotopic peaks corresponding to triply to hexaply charged ions were observed at *m*/*z* = 1491.2181, 1118.6656, 895.1341, and 746.1114. The major impurities observed were monooxidized species, proline adducts, and glycine adducts, which accounted for 13.9, 4.5, and 1.5% of the intensity of the quadruply charged ion of the peptide, respectively. It should be noted that oxidation may have occurred during electrospray ionization.

### Preparation of Vesicles

PC, CH, and DPPS were dissolved in chloroform, and SM was dissolved in ethanol at a concentration of 10 mM. The solutions were mixed at various ratios (Table 1) in glass tubes. The mixed solutions were then dried under a flow of nitrogen gas, followed by hydration using a 285 mM sucrose solution at lipid concentrations ranging from 0.1 to 15 mg/mL. Sucrose was used to prevent aggregation and to stabilize both the membranes and the peptide. This hydration step was carried out at 60 °C using a water bath with a minute of sonication. Subsequently, the suspensions were extruded through a 100 nm pore polycarbonate membrane (Cytiva, Marlborough, MA, USA) at 60 °C five times using a high-pressure extruder (Northern Lipids, Burnaby, Canada).

**Table 1:** Lipid Compositions of the Vesicles

sample name	DPPC %	CH %	SM %	DPPS %
DPPC	100	0	0	0
CH10	90	10	0	0
CH20	80	20	0	0
CH30	70	30	0	0
CH40	60	40	0	0
SM5	95	0	5	0
SM10	90	0	10	0
SM15	85	0	15	0
SM15CH10	75	10	15	0
SM15CH20	65	20	15	0
SM15CH30	55	30	15	0
SM15CH40	45	40	15	0
SM15CH40DPPS5	40	40	15	5

### Preparation of Peptide-Loaded Vesicles by the Incubation-Extrusion Method

A peptide with the integrin α transmembrane domain sequence was dissolved in a chloroform/methanol (9:1, v/v) solution at 1 mg/mL. The solution was then mixed with the lipids in organic solvent at a peptide concentration of 2 wt % relative to the lipids, followed by solvent removal, hydration, and extrusion as described above, except that the suspension was incubated at 25 °C for 24 h prior to extrusion.

### Preparation of Peptide-Loaded Vesicles by the CHAPS Method

A zwitterionic detergent, CHAPS, was used to load the peptide into the SM15CH40 membrane as an alternative method.^15–18^ The peptide was dissolved at 0.1 mg/mL in a solution containing 1 vol % CHAPS and 285 mM sucrose, which was then added to hydrated, but not yet extruded, SM15CH40, at 10 vol %, at a peptide concentration of 2 wt % relative to the amount of lipids. The suspension was incubated at 25 °C for 24 h, followed by five extrusion cycles at 60 °C. CHAPS was removed by diafiltration using an Amicon Ultra Centrifugal Filter with a molecular weight cutoff of 10,000 Da (Merck Millipore, Burlington, MA, USA). A control sample without the peptide was prepared by using the same procedure. The vesicle concentration was adjusted to 0.23 mg/mL based on the UV absorption at 215 nm.

### Dynamic Light Scattering (DLS)

DLS analysis was carried out using a Stunner system (Unchained Laboratories, Pleasanton, CA, USA) equipped with a 660 nm laser diode and a detector at 142°. 2 μL portion of vesicles diluted to 0.1 mg/mL with the sucrose solution was applied to a microfluidic circuit on a Stunner plate. Each measurement was repeated four times in duplicate or in triplicate for different samples to confirm the reproducibility. The Lunatic Client software (Unchained Laboratories) was used to analyze the data and provide *Z*-average diameters based on cumulant analysis. The viscosity and refractive index of the sucrose solution used for the calculation were 1.07 cP and 1.34, respectively.^19,20^

### Nanoparticle Tracking Analysis (NTA)

A NanoSight NS-300 system (Malvern Panalytical, Worcestershire, U.K.) equipped with a 488 nm laser was used to analyze the particle size distribution. For the measurements, vesicles prepared at 0.1 mg/mL were diluted 1000-fold with 285 mM sucrose solution, which was filtered through an Amicon Ultra Centrifugal Filter with a cutoff molecular weight of 10,000 Da beforehand. This filtration step was crucial for the analysis, as sucrose-derived particles were otherwise observed, as described in the Supporting Information (Figure S1). The measurements were carried out at 25 °C.

### Lipid Concentration Measurements

Lipid concentrations of the vesicles were measured by high-performance liquid chromatography (HPLC) on a Nexera XR system (Shimadzu, Kyoto, Japan), using an Inertsil ODS-4 as a separation column (250 × 4.6 mm^2^ ID, 3 μm particle size, GL Sciences, Tokyo, Japan). An isocratic flow with a mobile phase consisting of methanol, tetrahydrofuran, and 170 mM ammonium acetate solution (94:5:1, v/v/v) was used at a flow rate of 0.5 mL/min, where the column temperature was maintained at 35 °C. The lipids were detected using a photodiode array detector at a wavelength of 215 nm. Vesicles prepared at 1 mg/mL were diluted 5-fold with ethanol, and 10 μL was injected for analysis. A standard solution containing 1 mg/mL of DPPC, SM, and CH in 80 vol % ethanol was sequentially diluted with the same solvent to obtain a standard curve ranging from 0.0125 to 1 mg/mL, where linearity was confirmed.

### Differential Scanning Calorimetry (DSC)

DSC measurements were performed on a MicroCal PEAQ-DSC system (Malvern Panalytical) at a heating rate of 1.5 °C/min, with 5 min of isothermal stabilization before every run, and in high-feedback mode. A sucrose solution was used as the reference. Samples ranging from 5 to 15 mg/mL were subjected to measurements after vacuum treatment for approximately 5 min. The baseline of the signal was corrected using a spline curve to determine the peak-top phase-transition temperature (*T*_c_) and enthalpy (Δ*H*).

### Fluorescence Measurements to Determine Membrane Polarity

Laurdan, a fluorescent polarity probe, was dissolved in chloroform at a concentration of 1 mM and added to the lipid solutions at a final concentration of 1 mol % relative to the total lipid content. Vesicles were prepared under protection from light using the method described above. The probe was excited at 340 nm, and fluorescence spectra from 360 to 600 nm were acquired using an FP-6500 spectrofluorometer (Jasco Corp., Tokyo, Japan). The measurements were performed at 25 and 37 °C. Generalized polarization (GP) values^21,22^ were calculated from the fluorescent intensities at 440 and 490 nm using the following equation to quantify the relative polarity of the membranes:

1 $$\mathrm{GP}=\left(I_{440\mathrm{nm}}-I_{490\mathrm{nm}}\right)/\left(I_{440\mathrm{nm}}+I_{490\mathrm{nm}}\right)$$

### Fluorescence Lifetime Measurements to Evaluate Membrane Heterogeneity

Fluorescence lifetime of laurdan incorporated in 0.1 mg/mL vesicles at 1 mol % was measured by a FluoroCube time-correlated single-photon counting (TCSPC) system equipped with a PicoBrite pulse laser at 375 nm (HORIBA, Kyoto, Japan), with a suspension of LUDOX-HS-30 colloidal silica (Sigma-Aldrich) as a reference. The lifetime measurement was carried out at 25 and 37 °C, at reverse TCSPC mode, with a coaxial delay time of 85 ns, a synchronization delay time of 0 ns, and a repetition rate of 16 MHz. The measurement continued until the photon count reached 3000. Decay profile simulations were performed using a nonlinear least-squares method on the DAS6 fluorescence decay analysis software (HORIBA). The data was first fitted to a single-component decay function, *F*(*t*), as follows:

2 $${F\left(t\right)=A+Be}^{-t/T}$$

where *t* and *T* represent the time and the lifetime of the probe, respectively. *A* and *B* are constants. The number of exponentials (or decay components) was increased until *F*(*t*) fitted the data, and the χ^2^ value was below 1.2. For instance, the fluorescence decay of the two components is given by

3 $${F\left(t\right)=A+B_{1}e}^{-t/T_{1}}{+B_{2}e}^{-t/T_{2}}$$

where *T*_1_ and *T*_2_ are the lifetimes of the decay components, and *B*_1_ and *B*_2_ provide the relative amplitudes (percentages of photons coming from different decays) calculated by *B*_*i*_ *T*_*i*_/∑*B*_*i*_ *T*_*i*_. The fluorescence decay curve obtained from a homogeneous membrane is linear on a semilogarithmic plot, whereas that in a phase-separated membrane shows a nonlinear curve due to multiple exponential components. Fluorescence decay curves for DPPC, CH10, CH40, SM15, SM15CH10, and SM15CH40 membranes were obtained at 10 nm intervals in the range from 420 to 500 nm, at 25 and 37 °C.

### Atomic Force Microscopy (AFM)

The bending stiffness of the lipid membranes was determined by quantitative imaging using a NanoWizard ULTRA Speed atomic force microscope system (JPK, Berlin, Germany), where the cantilever was pressed at the center of a vesicle and the stress was measured.^23–25^ Bending stiffness of CH10, CH40, SM15, SM15CH10, and SM15CH40 was evaluated at room temperature. DPPC was excluded from the evaluation because of its aggregation. Aminopropylated mica substrates (AP-mica) were prepared by immersing mica substrates (SPI-Chem Mica grade V-1 12 mm D × 0.15 mm thickness, SPI Supplies, West Chester, PA, USA) in 1% 3-aminopropyltriethoxysilane for 20 min at room temperature, followed by rinsing with deionized water. Prior to the experiment, the mica substrate was glued onto a glass slide with an acrylamide ring as a barrier, allowing surface modification and a subsequent sample fixation process to be performed without direct contact with the mica substrate. Vesicles were fixed on the mica substrate by leaving 100 μL of 100 μM vesicles for 10 min at room temperature, followed by the addition of 1.4 mL of sucrose solution. A commercial silicon-based cantilever BioLever mini (BL-AC40TS-02, Olympus, Tokyo, Japan) of nominal spring constant 0.09 N/m was calibrated via the thermal-noise method prior to the imaging.^26^ The quantitative imaging was conducted over a 1 μm × 1 μm area at 128 pixel × 128 pixel (<8 nm/pixel), with a set point of 150–250 pN, cantilever speed of 15 μm/s, and *z*-resolution of 20 points/nm. Approximately 10 images were acquired per sample to capture 80–120 vesicles. Vesicle stiffness was analyzed as described previously.^27^

### ζ Potential Measurement

The ζ potentials of the vesicles were measured by using a Zetasizer Nano-ZS system (Malvern Panalytical). Samples were diluted to 0.01 mg/mL with 285 mM sucrose solution, which was filtered through an Amicon ultra centrifugal filter with a cutoff molecular weight of 10,000 Da (Merck Millipore) prior to use. A folded capillary ζ cell (Malvern Panalytical) was used for the measurements, and a voltage of 150 V was applied. Each measurement was performed with ten repeats, and data were analyzed using the Smoluchowski model with the refractive index and viscosity of the sucrose solution described earlier. Particle size measurements were also performed on the same system for making comparisons with the data obtained by NTA. The measurement was repeated four times, with duplicates per sample to confirm reproducibility. The detection angle was 173°.

### CD Spectroscopy

CD spectra of the peptides incorporated into the membranes were obtained by using a J-815 CD spectrometer equipped with a temperature controller PTC-423S (Jasco, Tokyo, Japan). The samples were diluted to 0.23 mg/mL with a 285 mM sucrose solution. A quartz cell with an optical path length of 10 mm was used for the measurements. CD spectra between 200 and 260 nm were measured at 25 °C, at a scan speed of 50 nm/min, with a response speed of 4 s at standard sensitivity. The spectral data were averaged over 12 times to eliminate noise and obtain smooth curves. Nitrogen gas was introduced into the sample room at a flow rate of 10 L/min. A baseline curve was first recorded using lipid vesicles without the peptide. This baseline was then subtracted from the spectrum of vesicles containing the peptide.

## Results

### Effect of Extrusion Process on Lipid Composition and Concentration of Vesicles

The lipid concentrations of SM15CH40 and SM15CH10 before and after extrusion were measured by using HPLC to verify that slight clogging during extrusion did not affect the final lipid composition and concentration. After five extrusion cycles, the total peak area of SM15CH40 decreased by 7% and that of SM15CH10 decreased by 9% (data not shown), which would not significantly affect the results of subsequent experiments. In addition, the lipid compositions of both samples remained mostly unchanged (Figure S2A–C).

### Size Distribution of Phospholipid Vesicles

The influence of SM, CH, and negatively charged PS on the size distribution of the phospholipid vesicles was investigated by using NTA. DPPC vesicles were not evaluated because they aggregated immediately after preparation. All samples had mean diameters of approximately 90–100 nm, with slightly larger diameters observed for those containing 40 mol % CH (Figure 1A–F). The addition of charged DPPS did not affect the vesicle size distribution (Figure 1F). As the vesicle size was controlled during the extrusion, the difference in size originates from the difference in the membrane stiffness and possible fusion of vesicles after the extrusion. DLS was also employed for particle size measurements to confirm whether differences in the measurement principle influenced the results. When analyzed by DLS, all vesicles exhibited larger average sizes than the mean and mode diameters obtained by NTA (Table 2). NTA is a number-based evaluation method that determines the velocity of Brownian motion for each particle, whereas DLS calculates the average diameter, an intensity-based average particle size, from the fluctuation in the scattered light intensity of the entire sample solution. The average diameter tends to be biased toward larger particle sizes. The result is also easily influenced by the presence of larger particles, because the scattered light intensity is proportional to the sixth power of the particle diameter. When the vesicles were visualized by AFM, the mean diameters were closer to the results of NTA than to those of DLS (Table 2 and Figure S2A–E).

**Figure 1:**
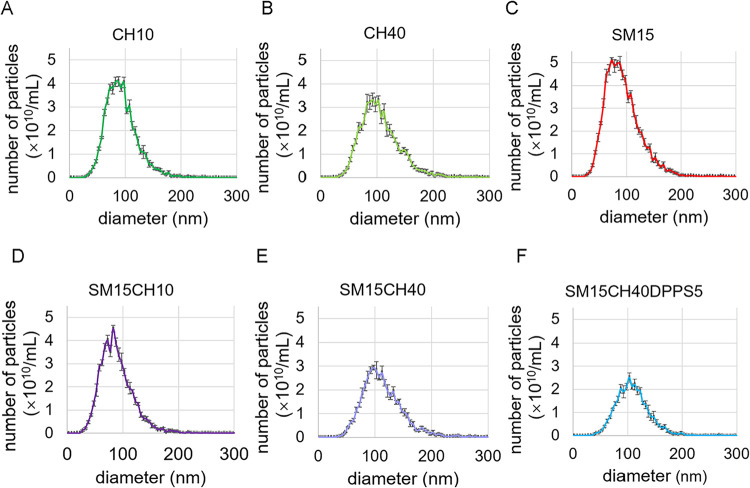
Size distribution of 0.1 mg/mL vesicles analyzed by NTA. (A) CH10, (B) CH40, (C) SM15, (D) SM15CH10, (E) SM15CH40, and (F) SM15CH40DPPS5.

**Table 2:** Comparison of NTA, DLS, and AFM Data on the Size Distribution of the Vesicles

	NTA			DLS		AFM		AFM	
	mean (nm)	mode (nm)	SD	average diameter (nm)	PDI	mean diameter (nm)	SD	mean height (nm)	SD
CH10	93.6	85.4	29.1	109	0.10	82.4	21.4	69.3	17.8
CH40	106	91.8	36	129	0.14	113	27	75.6	18.1
SM15	92.7	73.2	32.3	111	0.11	116	27	72.2	18.0
SM15CH10	89.0	83.1	29.8	109	0.15	75.7	17.4	73.1	19.0
SM15CH40	114	97.5	37	125	0.14	113	27	69.8	19.5

### Effect of SM and CH on Thermodynamic Behaviors of the Membrane

The DSC measurements revealed that the addition of SM to the DPPC membrane decreased the phase-transition temperature (*T*_c_) and increased the phase-transition enthalpy (Δ*H*), whereas the addition of CH broadened the transition peak to reduce Δ*H* (Table 3 and Figure 2A–D). The addition of 40 mol % CH resulted in a 90% loss of Δ*H*, indicating that the change in the physicochemical properties at the transition temperature became less clear, which agrees with the previous reports.^28^ The addition of SM to DPPC at 15 mol % increased Δ*H* by 37%, which was also in agreement with prior literature on DPPC/palmitoyl sphingomyelin (PSM) mixture,^29^ where a slight increase in Δ*H* was observed as the PSM ratio increased up to 40%. The reduction in Δ*H* by CH was partially recovered by the addition of SM, indicating that these molecules have opposite roles in the phase-transition properties of the membrane.

**Figure 2:**
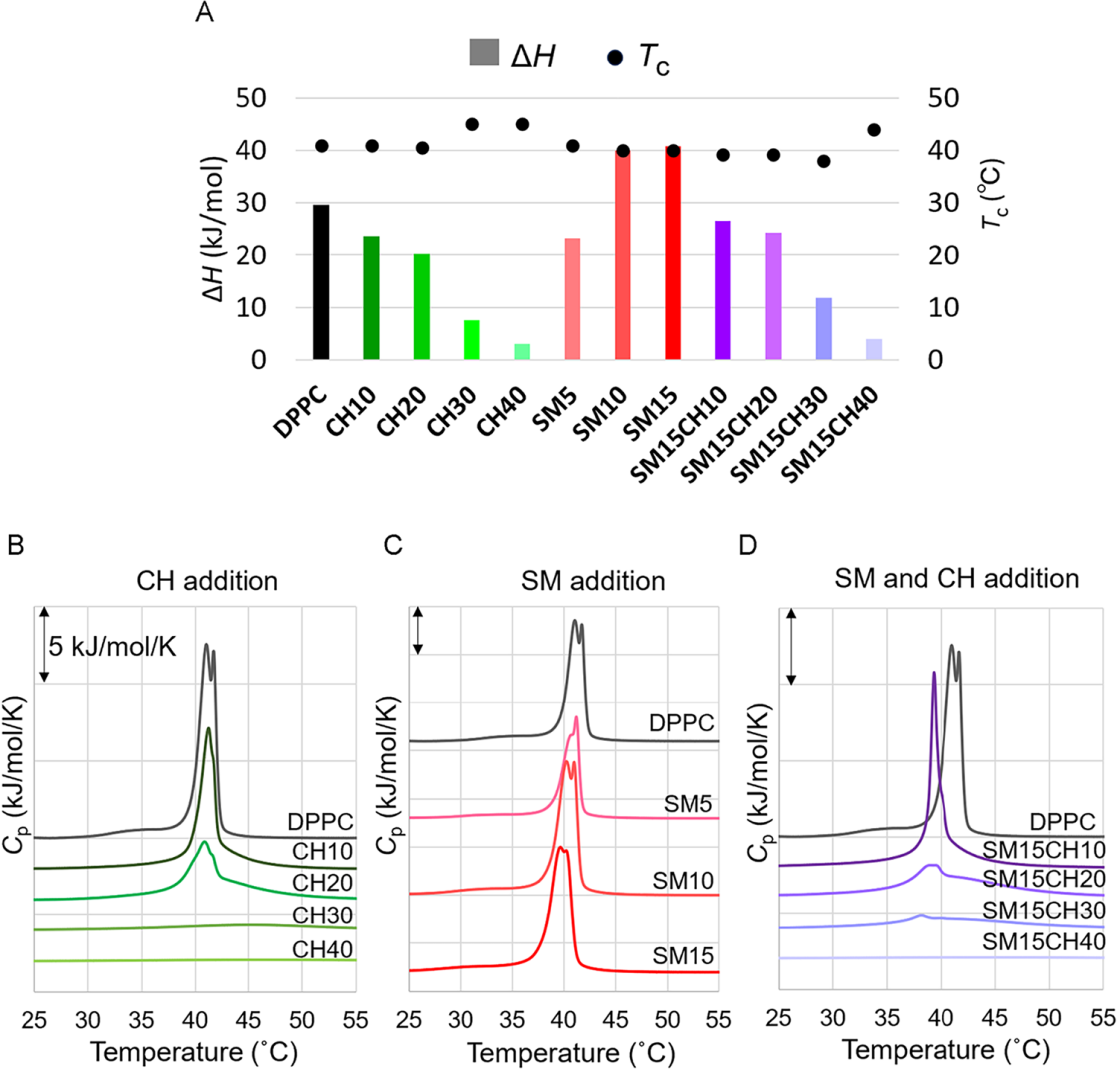
(A) Phase-transition enthalpy (bar graph) and temperatures (dots) of lipid membranes with different compositions. (B–D) DSC curves showing the impact of (B) CH addition, (C) SM addition, and (D) both CH and SM addition to DPPC. CH30, CH40, and SM15CH30 were prepared and measured at 10 mg/mL, and SM15CH40 was measured at 15 mg/mL. Other vesicles were prepared and measured at 5 mg/mL.

**Table 3:** Phase-Transition Enthalpy and Temperature

sample	Δ*H* (kJ/mol)	*T*_c_ (peak top) (°C)
DPPC	29.4	41.0
CH10	23.7	41.0
CH20	20.3	40.5
CH30	7.60	45.0
CH40	3.00	45.0
SM5	23.1	41.0
SM10	39.8	40.0
SM15	40.5	40.0
SM15CH10	26.5	39.2
SM15CH20	24.4	39.2
SM15CH30	12.0	38.0
SM15CH40	3.91	44.0

The vesicles were prepared at higher concentrations for DSC measurement than those for other evaluations, as it was required for assuring sensitivity. This likely influenced the DSC curves of some samples to exhibit split peaks, suggesting the coexistence of unilamellar and multilamellar vesicles. Nevertheless, the overall enthalpy of the DPPC vesicles was similar to the one reported for large unilamellar vesicles.^28^

### Effect of SM and CH on Membrane Polarity

The fluorescence spectrum of laurdan incorporated into the DPPC membrane is known to peak at approximately 440 nm below *T*_c_, which is weakened and shifts to 490 nm with increasing temperature as the membrane undergoes a phase transition.^30^ The red shift in the laurdan spectrum indicates an increase in polarity,^31^ which may be explained by the diffusion of water molecules into the membrane.^22^ Fluorescence intensities at 440 and 490 nm were used to calculate the GP values, as described in the Methods. A higher GP value indicates a lower polarity that originated from tighter packaging of the lipid membrane. Figure 3A–F shows the impact of CH and/or SM addition on the fluorescence spectra of laurdan, at 25 and 37 °C. The polarity at 37 °C was higher than that at 25 °C, regardless of the membrane composition (Figure 3G). The effects of CH and/or SM addition on the membrane polarity were clearer at 37 °C than at 25 °C, as it was closer to *T*_c_. At 37 °C, CH and SM decreased and increased the polarity, respectively, while they both decreased the polarity at 25 °C. The polarity change between 25 and 37 °C was suppressed in the presence of CH but enhanced in the presence of SM, which is consistent with the trend of Δ*H*. The membrane polarity depends on the lipid packing, which is influenced by membrane fluidity. In the presence of CH, the contrast above and below the transition is smaller than that for pure DPPC membrane because of the increase in fluidity in the gel state and its decrease in the liquid state. The contribution of SM is likely to be the opposite. As the membrane assembly is dominated by hydrophobic interaction, the ordering of the hydrophilic part, including hydrated water molecules, should make a significant contribution to Δ*H*. As this is also influenced by the change in membrane fluidity at the transition temperature, it seems to be natural for the polarity and Δ*H* to exhibit the same trend.

**Figure 3:**
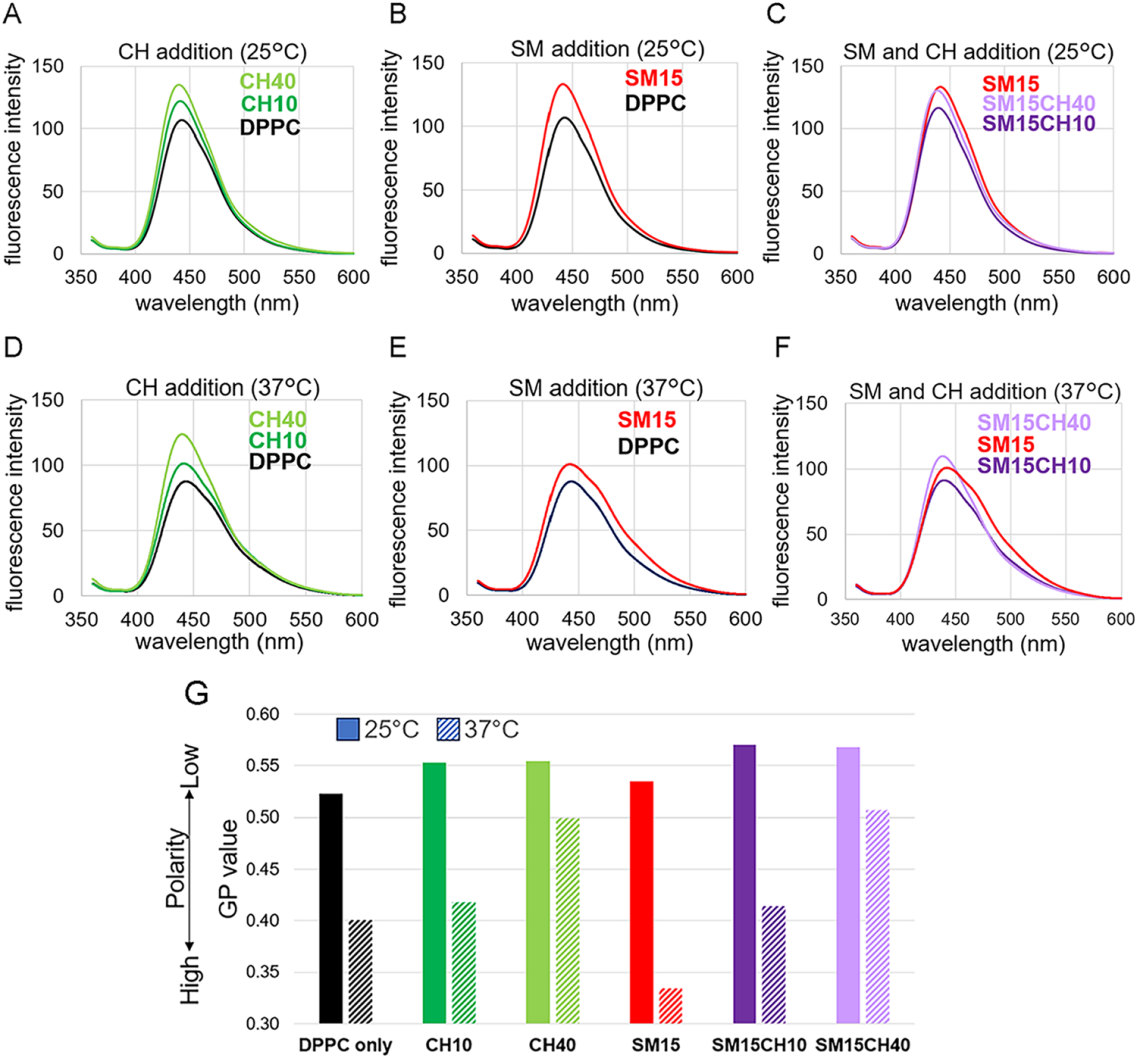
Effect of CH and SM on the fluorescent spectra of laurdan and the membrane polarity. Fluorescence spectra of laurdan showing the impact of (A) CH addition at 25 °C, (B) SM addition at 25 °C, (C) SM and CH addition at 25 °C, and (D–F) their counterparts at 37 °C. (G) GP values of the membranes at 25 and 37 °C.

### Dynamic Heterogeneity of the Lipid Membrane Revealed by Fluorescence Lifetime Analysis

Figure 4 shows the fluorescence lifetime of laurdan and the proportion of lifetime components in different membranes at 25 and 37 °C. Only one component with 6.0–7.3 ns lifetime representing gel-like phase (gel or l_o_ with lower mobility) was detected for DPPC, CH10, and CH40 membranes at any wavelength at 25 °C, whereas a second component with a short fluorescence lifetime of 3.0–3.3 ns appeared at 460–470 nm for SM15, SM15CH10, and SM15CH40 (Figure 4A and Tables S1 and S2). The short fluorescence lifetime of the second component indicates loose packing and high molecular mobility of the surrounding lipids that should facilitate quick energy transfer from the excited fluorophore to the ground state. The proportions of the second component were estimated to be 0.8% for SM15, 0.6% for SM15CH10, and 1.2% for SM15CH40 (Figure 4B), relative to the area under the curve (AUC) of the fluorescent spectra of laurdan within the 420–500 nm range.

**Figure 4:**
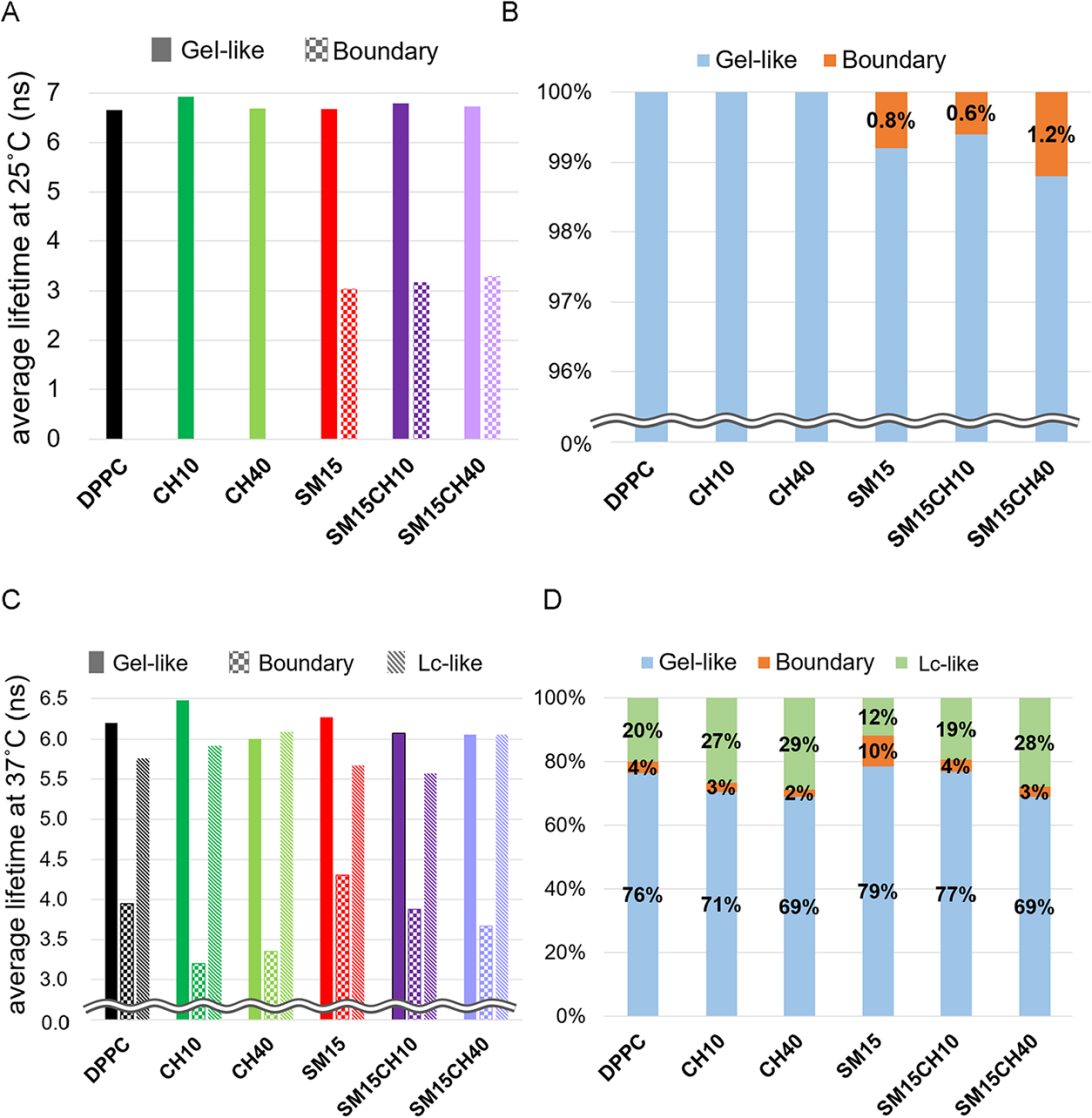
Fluorescence lifetime of laurdan and its proportion in different membranes at 25 and 37 °C. (A) Average fluorescence lifetimes of the gel-like and the boundary phase at 25 °C and (B) their percentages. (C) Average fluorescence lifetimes of the gel-like phase, the boundary phase, and the l_c_-like phase at 37 °C in different membranes and (D) their percentages.

At 37 °C, the presence of three components was confirmed for all membranes tested (Figure 4C). These components had the fluorescence lifetimes of 5.5–7.3, 3.2–4.5, and 4.9–6.2 ns, and were observed in a relatively shorter, intermediate, and longer wavelength ranges, respectively (Tables S3–S5). Based on the polarities indicated by the wavelength ranges and the molecular packing indicated by the fluorescence lifetime, the first and the third components were understood to be the gel-like and liquid-crystalline (l_c_)-like (l_c_ or l_o_ with higher mobility) phases, respectively. Addition of 40% CH diminished the difference in average fluorescence lifetimes between the gel-like phase and the l_c_-like phases, while 15% SM enhanced it, which was consistent with the tendencies observed for changes in Δ*H* and GP. The second component, which had the shortest lifetime of laurdan and was detected at the intermediate wavelength range, was considered to reflect the boundary between the gel-like and the l_c_-like phases, since the interface between two distinct phases is inherently unstable, and such instability is associated with loose molecular packing and increased molecular mobility. The second component of 3.0–3.3 ns lifetime observed at 25 °C was also regarded as the boundary, although the l_c_-like phase was undetected, likely because its abundance was below the detection limit. Figure 4D shows the proportion of these three components calculated relative to the AUC of the fluorescence spectra of laurdan within the 420–450 nm range. SM was revealed to induce the boundary under both temperature conditions.

### Bending Stiffness of Lipid Vesicles Determined by AFM

Figure 5 shows the bending stiffness of the vesicles evaluated by AFM. The addition of 40% CH reduced the stiffness, which is consistent with the literature reporting the formation of a moderately fluid l_o_ phase at higher CH levels.^32,33^ On the other hand, the impact of the addition of SM on the bending stiffness was obscure. Although the bending stiffness of DPPC vesicles was not available because of their poor dispersion stability, it is expected to be between CH10 and SM15, considering other physical properties, including thermodynamic transition behavior and polarity. CH40 and SM15CH40 were found to have significantly softer membranes compared to those of other vesicles.

**Figure 5:**
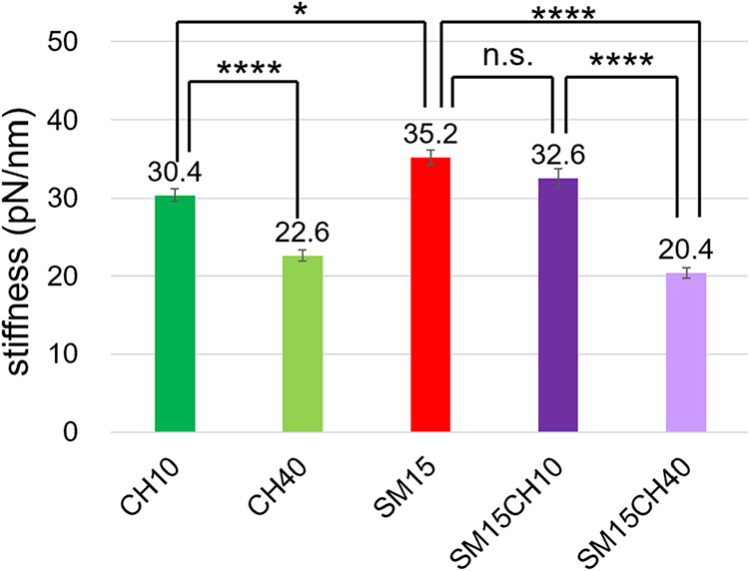
Bending stiffness of vesicles measured by AFM. Data are presented as mean ± standard deviation (SD). The statistical significance was determined by Tukey’s multiple comparison test. *p < 0.05, ****p < 0.0001, n.s.: not significant (*p* > 0.05).

### Peptide Constitution to the Vesicles

The transmembrane helical peptide of integrin α was loaded into the lipid membranes simply by mixing it with lipids in an organic solvent, followed by solvent removal, hydration, and extrusion. Figure 6A shows the effect of incubation before high-pressure extrusion on the secondary structure of the peptide, where an α-helical CD spectrum was observed in the incubated sample. The formation of the α-helical structure indicated successful constitution of the peptide in the lipid membranes. When extruded without incubation, the CD spectrum did not exhibit a 209 nm local minimum or a 222 nm local minimum, which are the characteristics of the α-helical structure.^34^ A successful α-helix formation of the peptide was achieved when the peptide/SM15CH40 mixture was incubated for 24 h at 25 or at 4 °C prior to extrusion but not when incubated at 40 °C (Figure 6B). When the peptide/SM15CH40 mixture was extruded without incubation and stored at 4 °C for 6 days, the CD spectrum did not change from the initial state (Figure 6C), suggesting that the mechanical stress after peptide-membrane interaction enhances peptide insertion into the membrane. The peptide concentration was set at 2 wt % to the vesicles in this investigation because the sensitivity of CD measurement was insufficient at concentrations lower than 2%, and the addition of 4% peptide caused aggregation of vesicles (data not shown). Surfactants such as Triton X-100 or CHAPS are commonly used when peptides or proteins are incorporated into membranes. Consequently, we also tested a method in which the peptide solubilized in the CHAPS solution was added to the SM15CH40 vesicle solution, followed by incubation, extrusion, and diafiltration. The diafiltration step was performed to remove the remaining CHAPS and unincorporated peptide molecules. However, the sample obtained by this method did not exhibit an α-helical CD spectrum (Figure S4).

**Figure 6:**
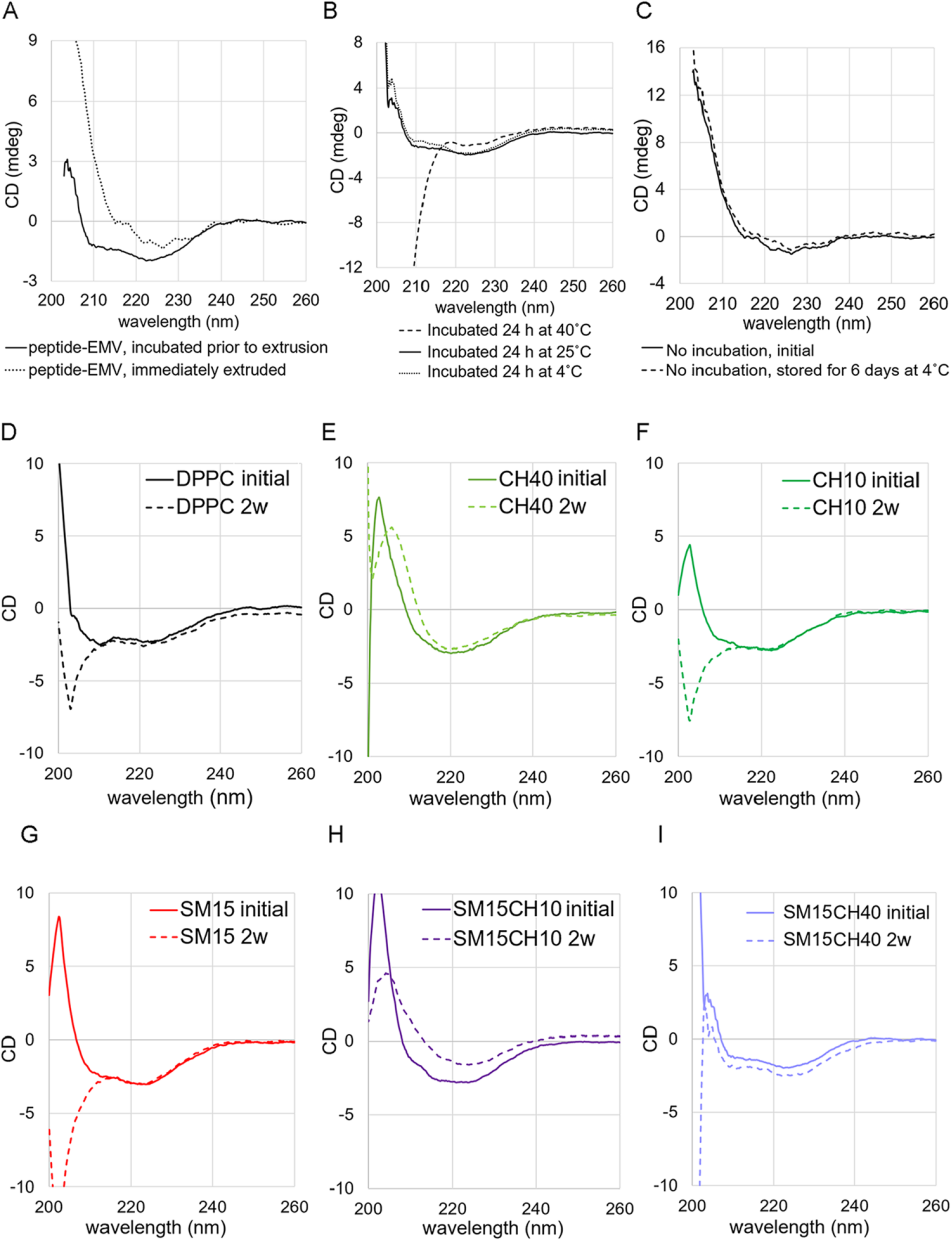
CD spetra of integrin transmembrane peptide in EMV (A) incubated or not incubated before extrusion, (B) incubated at different temperatures before extrusion, and (C) without incubation on the first day and after 6 days of storage at 4 °C. (D–I) CD spectra of constituted peptide in different membranes at initial time point and after 2 weeks of storage at 4 °C.

Lipid composition also affected the secondary structure of the peptide. In vesicles with less SM or CH, the CD spectra exhibited similar but different patterns that cannot be elucidated as that of α-helical structure, at the initial time point (Figures 6D–I and S5A–C). Interestingly, CD spectra obtained after 2 weeks of storage at 4 °C revealed that the secondary structure of the peptide was stable only in SM15CH40, not in the other vesicles.

Figure 7 and Table 4 describe the ζ potential of the peptide-loaded vesicles and the peptide-free vesicles evaluated after 2 weeks of storage at 4 °C. Proper insertion of the peptide should not cause a significant change in the ζ potential because most region of the molecule is entrapped in the lipid membrane. Peptide-bearing CH10 and SM15 exhibited positive charges, indicating that the cationic peptide was not properly entrapped in the membrane but was either adsorbed on the surface or protruding from the vesicles. CH40 with the peptide exhibited a more negative charge than peptide-free CH40, suggesting that the terminal anionic amino acid protruded from the membrane.

**Figure 7:**
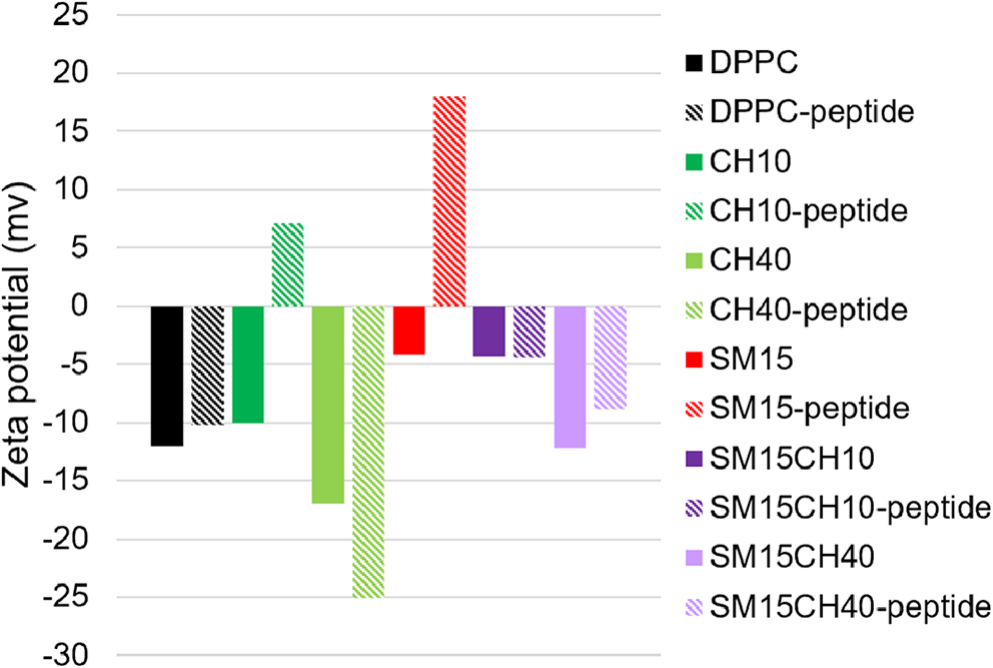
ζ potential of vesicles with or without the peptide.

**Table 4:** ζ Potential, the Average Diameter, and the PDI of the Vesicles with or without the Peptide

	ζ potential (mV)	average diameter (nm)	PDI
DPPC	–12.0	124	0.13
DPPC-peptide	–10.2	116	0.32
CH10	–10.0	112	0.15
CH10-peptide	7.11	118	0.22
CH40	–17.0	123	0.12
CH40-peptide	–25.1	131	0.22
SM15	–4.15	106	0.17
SM15-peptide	21.8	86.8	0.23
SM15CH10	–3.31	120	0.14
SM15CH10-peptide	–4.39	121	0.27
SM15CH40	–12.1	125	0.11
SM15CH40-peptide	–8.85	133	0.21

Although the peptide constitution did not significantly affect the average particle size of the vesicles (Table 4), aggregation was observed for most peptide-loaded vesicles except for SM15 and SM15CH40 after 2 weeks of storage at 4 °C. These results suggest that both SM and CH are essential for the successful and stable constitution of this helical peptide. The polydispersity index (PDI) values for the peptide-loaded vesicles were higher than those of the peptide-free vesicles, indicating a stronger attractive interaction between the peptide-loaded vesicles.

## Discussion

### Effect of CH and SM on the Physicochemical Properties of Lipid Membrane

DPPC membrane undergoes phase transition from gel (or solid-ordered (s_o_) phase) to liquid crystalline (or liquid-disordered (l_d_) phase) at its main transition at 41 °C. Rippled phase, which is the coexistence of s_o_ and l_d_ aligned periodically, is formed at a temperature between the pretransition at 37 °C and the main transition.^32,35,36^ Addition of 10–30 mol % CH to DPPC induces coexistence of s_o_ and l_o_ phase below *T*_c_,^26^ and further addition of CH (30–50 mol %) provides a homogeneous l_o_ phase that does not exhibit significant state change upon temperature increase.^37,38^ In simpler words, CH renders the gel-phase more fluid and the liquid-crystalline phase more rigid.^22^ Thus, CH addition reduces Δ*H*,^39–41^ which is consistent with our observation. It should be noted that the Δ*H* of the DPPC membrane is influenced by lamellarity,^28,42^ and our enthalpy data are similar to those reported for 100 nm large unilamellar vesicles,^28^ although split peaks indicating heterogeneous lamellarity were observed for some samples.

Although the effects of CH addition on PC membranes have been widely investigated, knowledge of the effects of SM addition is limited. The addition of 40 mol % CH to egg SM membrane was reported to decrease Δ*H* from 5.8 kcal/mol (24.3 kJ/mol) to 0.5 kcal/mol (2.1 kJ/mol), where the transition peak broadened and shifted toward higher temperature.^43^ A similar observation was reported for CH addition to the PSM membrane.^29^ These observations suggest that the effect of adding CH to the SM membrane is similar to that of its addition to the PC membrane. Meanwhile, the addition of PSM to the DPPC membrane slightly increased Δ*H* below 40% PSM.^29^ The phase diagram of DPPC/SM/CH ternary mixture has not been reported, while that of palmitoyloleoylphosphatidylcholine (POPC)/PSM/CH is available^44^ According to the phase diagram, s_o_ and l_o_ phases appeared upon addition of PSM and CH to POPC, respectively, under the temperature region where pure POPC membrane forms the l_d_ phase. At 15% PSM and 40% CH, the coexistence of l_d_ and l_o_ is described at both 25 and 37 °C. The DPPC membrane forms the s_o_ phase at room temperature. Given the structural and physicochemical similarities between DPPC and SM, we expected the phase behavior of the DPPC/SM/CH mixture to resemble that of the DPPC–CH mixture. Our physicochemical investigations indicated that SM15CH40 was indeed similar to CH40 in many aspects, including the thermodynamic behavior, the polarity, and the stiffness. The fluorescence lifetimes of the gel- and l_c_-like phases of CH40 and SM15CH40 were also similar, showing little temperature-dependent change. Therefore, the majority of SM15CH40 is considered to be in the l_o_ phase. The only difference between CH40 and SM15CH40 was the appearance of a small fraction of the boundary phase at 25 °C for SM15CH40.

The difference in the GP values between 25 and 37 °C was smaller and larger in the presence of CH and SM, respectively, than that for the pure DPPC membrane (Figure 3G). A similar trend was observed for the change in the Gibbs energy calculated from the DSC curves (Figure 8), which suggested a relevance between the polarity and thermodynamic stability of the lipid membranes. This may be a natural finding, as both are affected by the molecular packing of the lipid membrane.

**Figure 8:**
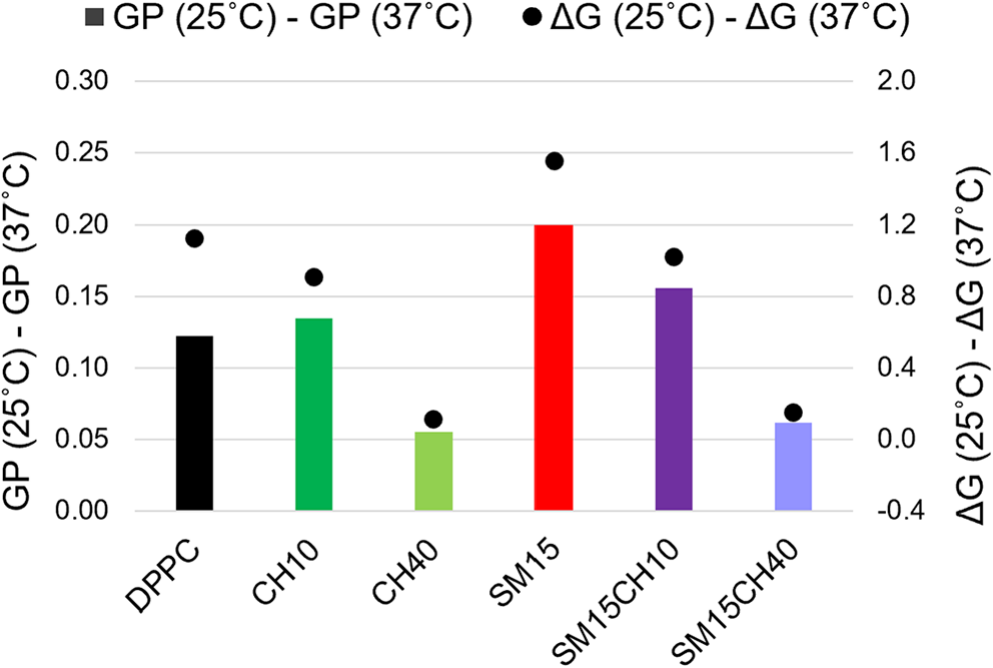
Comparison of the differences in GP (bar graph) and Δ*G* values (dots) obtained at 25 and 37 °C.

Decrease in the membrane polarity by CH addition at both 25 and 37 °C is consistent with a previous report,^22^ in which the effect was explained by (i) the displacement of water molecules by CH and (ii) tight packing of phospholipids as a consequence of a restriction of their molecular motions. The effect of the SM on the membrane polarity can be explained in the same manner. Importantly, the fluorescence spectra of laurdan incorporated in several cell membranes are reported to be similar to those in CH-rich DPPC/CH membranes, indicating a dominant role for CH in influencing membrane polarity.^45,46^ Thus, our exosome-mimetic membrane (SM15CH40) is expected to offer an environment similar to that of exosome membranes.

Fluorescence lifetime analysis revealed the presence of three components at 37 °C for all of the vesicles. Two of them could be easily assigned to the gel-like and l_c_-like phases based on the emission wavelength of laurdan. The lifetime of laurdan in DPPC membrane in its gel-phase is reported to be 5.9 ns, while that for 1,2-dilauroyl-sn-glycero-3-phosphocholine (DLPC) membrane in the l_c_-phase is 4.0 ns.^47^ In our data, the gel-like and l_c_-like phases had fluorescent lifetimes of 6–7 and 5–6 ns, respectively, showing a trend consistent with the previous report. The lifetimes of the gel-like phase and the l_c_-like phase in the CH-rich membranes were intermediate between those of the DPPC membrane (Figure 4C), because CH rendered the gel-like phase more fluid and the l_c_-like phase more solid. The impact of 15% SM is likely the opposite of CH, as assumed from the fluorescence lifetimes and the Δ*H*. The short-lifetime component, which was detected at an intermediate wavelength, suggested high mobility and might be interpreted as the boundary between the gel-like and the l_c_-like phases. Such an intermediate fluorescence spectrum has been observed in membranes composed of gel and l_c_, although detailed discussion has not been available.^47,48^

### Impact of the Physicochemical Properties on the Constitution of Helical Peptide

The presence of the highly mobile boundary phase in the SM15CH40 membrane at room temperature is likely one of the key factors for the successful constitution of the peptide, as it was the major difference between CH40 and SM15CH40. Peptide loading had no significant impact on the particle size or dispersion stability of SM15CH40, but induced aggregation of CH40, indicating the importance of the boundary phase induced by SM. However, the presence of the boundary phase alone was insufficient for the successful constitution of the peptide. CH was also required to maintain the peptide helicity within the membrane. AFM study revealed that the addition of CH increased the softness of the membrane. CH-induced fluidity/softness also seems to play an important role in tolerating and maintaining the α-helix structure within the membrane. Importance of CH on peptide insertion is also reported for amyloid-β, where molecular dynamics simulation analysis revealed that the incorporation of CH into the phospholipid bilayer increases surface hydrophobicity and alters lipid packing to enhance amyloid-β monomer binding to the CH-rich region of the membrane.^49^

Proper loading of the integrin transmembrane peptide into the SM15CH40 membrane required incubation before extrusion. This indicates that the insertion process of the peptide into the lipid membrane is slow, which is better assisted by mechanical stress to complete the proper folding into the α-helix in the membrane. Such time-dependent peptide insertion has also been reported for some amphiphilic peptides, including magainin 2, where the positive charge of the peptide enhances the electrostatic interaction with the phospholipid headgroup, followed by hydrophobic interaction with the acyl chains.^50,51^ Although the peptide was mixed with the lipids in the organic solvent in this study, it seems that the peptide was not immediately and fully integrated into the membrane in an α-helical conformation upon hydration of the dry film of the lipid-peptide mixture, as indicated by the importance of the incubation procedure prior to extrusion. The peptide constituted into the SM15CH40 membrane with the help of zwitterionic surfactant CHAPS did not exhibit an α-helical CD spectrum. This could be due to the residual surfactant, which likely altered the physicochemical properties of the membrane. Thus, our surfactant-free procedure appears to be more promising for the constitution of helical peptides and should also be applicable to integrin loading.

## Conclusions

The effects of SM and CH on the physicochemical properties of DPPC-based EMV were investigated, and their relevance to the constitution efficiency of a transmembrane helical peptide was examined. While SM enhanced temperature-dependent changes in the physicochemical properties and induced a small fraction of the highly mobile boundary phase at room temperature, CH suppressed temperature-dependent changes in the physicochemical properties and increased the softness of the vesicles. The constitution of the peptide was most successful with vesicles bearing 15% SM and 40% CH, and the exclusion of SM or CH resulted in low dispersion stability or unsuccessful peptide constitution. This was likely due to the requirement of both CH-induced membrane softness and the presence of the SM-induced boundary phase. Also, the α-helix structure formed efficiently by incubating the peptide-vesicle suspension before extrusion, which indicated that the spontaneous interaction between the membrane and the peptide, followed by mechanical assistance at high temperature, was important. These results provide important insights that serve as a foundation for developing EMVs as drug carriers.

## Supplementary Material



## Notes

Images of the impurity particles in sucrose solution observed by a nanoparticle tracking analysis system (Figure S1); chromatograms and a bar plot of peak areas from the quantification of lipids by liquid chromatography (Figure S2); images of the lipid vesicles obtained by AFM (Figure S3); fluorescence spectra of polarity probe laurdan incorporated in various lipid membranes (Figure S4); and CD spectra of a peptide incorporated in the lipid membranes at various conditions (Figures S5 and S6) (PDF)
