# Supplementary material for: Impact of Lipid Bilayer Composition and Physicochemical Properties on Constitution of a Transmembrane Helical Peptide into Exosome-Mimetic Vesicles
Source: Mol Pharm. 2025 Oct 8;22(11):6874–86. doi: 10.1021/acs.molpharmaceut.5c00825 (PMC12587397; doi:10.1021/acs.molpharmaceut.5c00825)
Supplement: Supplementary file 1 [file mp5c00825_si_001.pdf]

Supplementary Information

Impact of Lipid Bilayer Composition and Physicochemical Properties on Constitution of a Transmembrane Helical Peptide into Exosome-Mimetic Vesicles

Shiho Tsutsumi[1,2], Yuki Takechi-Haraya[3], Yasuhiro Abe[4], Kohsaku Kawakami[1,2,*]

[1] Research Center for Macromolecules and Biomaterials, National Institute for Materials Science, 1-1 Namiki, Tsukuba, Ibaraki 305-0044, Japan

[2] Graduate School of Science and Technology, University of Tsukuba, 1-1-1 Tennodai, Tsukuba, Ibaraki 305-8577, Japan

[3] Division of Biochemistry, National Institute of Health Sciences, 3-25-26, Tonomachi, Kanagawa, Kawasaki, 210-9501, Japan

[4] Division of Drugs, National Institute of Health Sciences, 3-25-26, Tonomachi, Kanagawa, Kawasaki, 210-9501, Japan

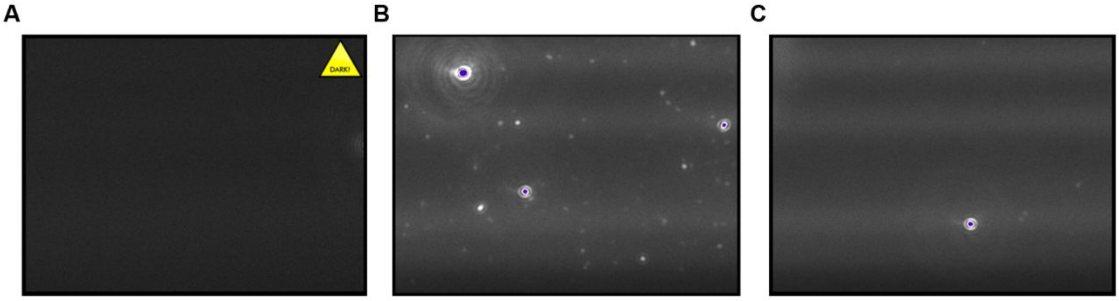

Fig.S1 NTA images of (A) MilliQ water (B) 285 mM sucrose solution without filtration (C) 285 mM sucrose solution after filtered through Amicon 10K device.

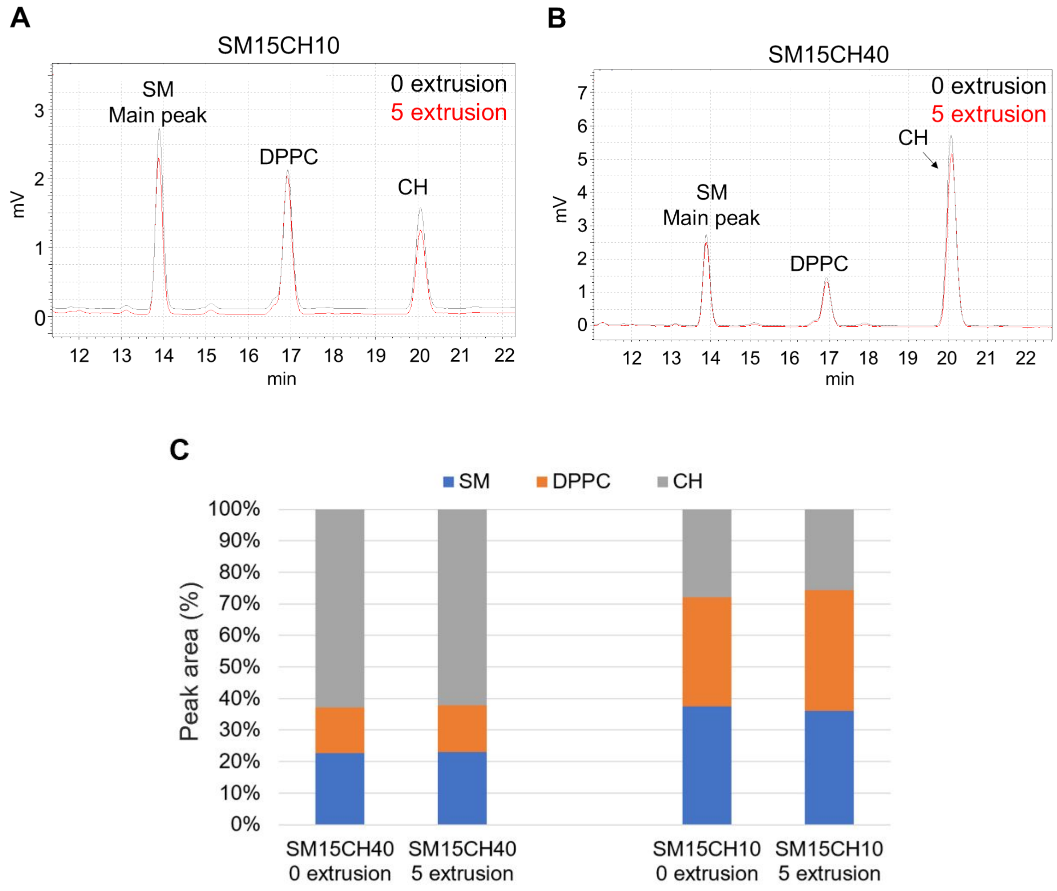

Fig.S2 (A) (B) Chromatograms of (A) SM15CH40 and (B) SM15CH10 vesicles with or without extrusion. (C) Peak area% of DPPC, SM, and CH in vesicles before and after extrusion. These data indicated that the slight clogging during extrusion did not significantly affect the final lipid concentration and composition.

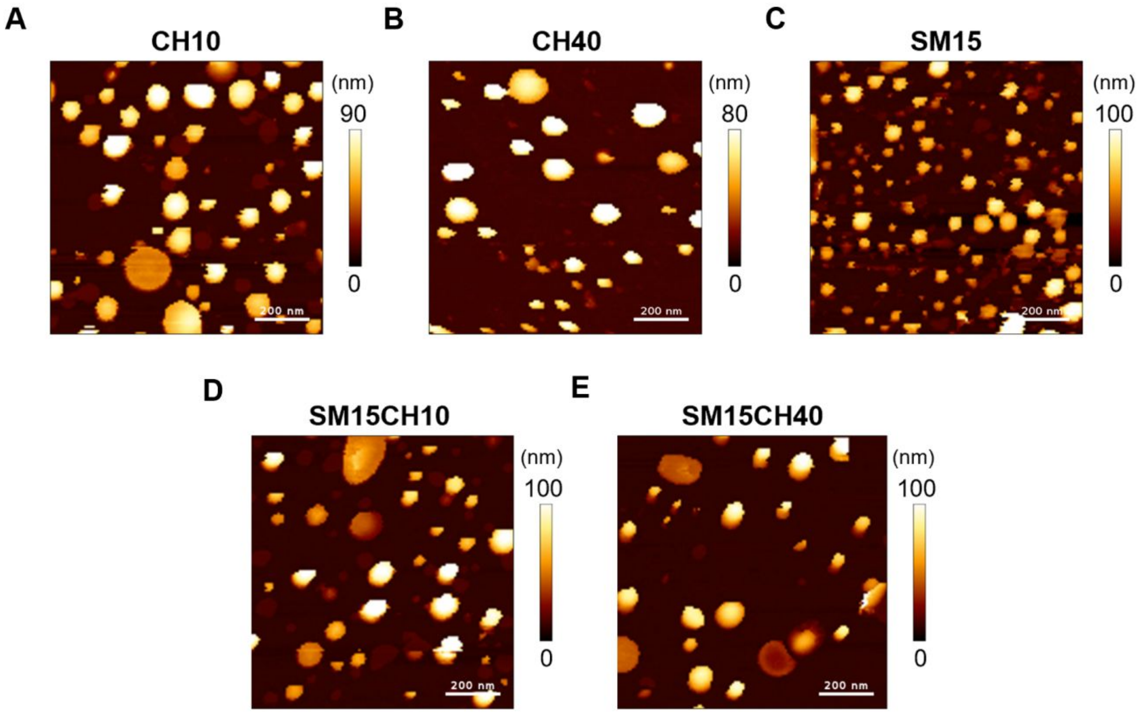

Fig.S3 AFM images of (A)CH10, (B) CH40, (C) SM15, (D) SM15CH10, and (E) SM15CH40.

Table S1 Fluorescence lifetime of laurdan in the gel-like phase at 25°C.

Wavelength (nm)	Fluorescence lifetime of laurdan in gel-like phase, 25°C (ns)					
	DPPC	CH10	CH40	SM15	SM15CH10	SM15CH40
420	6.13	6.43	5.97	6.20	6.27	6.21
430	6.53	6.57	6.53	6.43	6.53	6.46
440	6.56	6.78	6.58	6.53	6.66	6.59
450	6.68	6.91	6.72	6.70	6.79	6.76
460	6.78	7.06	6.82	6.78	6.86	6.94
470	6.61	6.93	6.75	6.91	7.01	6.73
480	6.81	7.05	6.92	6.73	6.91	6.92
490	6.84	7.20	6.93	6.83	7.03	6.97
500	6.92	7.34	6.93	6.93	6.98	6.96
	Average (ns)					
	6.65	6.92	6.68	6.67	6.78	6.73

Table S2 Fluorescence lifetime of laurdan in the boundary phase at 25°C.

Wavelength (nm)	Fluorescence lifetime of laurdan in boundary phase, 25°C (ns)					
	DPPC	CH10	CH40	SM15	SM15CH10	SM15CH40
420						
430						
440						
450						
460						3.30
470				3.04	3.18	
480						
490						
500						

Table S3 Fluorescence lifetime of laurdan in the gel-like phase at 37°C.

Wavelength (nm)	Fluorescence lifetime of laurdan in gel-like phase, 37°C (ns)					
	DPPC	CH10	CH40	SM15	SM15CH10	SM15CH40
420	5.70	6.12	5.67	5.73	5.84	5.72
430	6.06	6.27	5.74	5.95	6.08	5.90
440	6.20	6.36	6.11	6.06	6.02	6.05
450	6.22	6.3	6.19	6.11	6.05	6.19
460	6.40	6.49	6.31	6.26	6.11	6.42
470	6.64	7.34		6.89	6.36	
480				6.91		
490						
500						
	Average (ns)					
	6.20	6.48	6.00	6.27	6.08	6.06

Table S4 Fluorescence lifetime of laurdan in the boundary phase at 37°C.

Wavelength (nm)	Fluorescence lifetime of laurdan in boundary phase, 37°C (ns)					
	DPPC	CH10	CH40	SM15	SM15CH10	SM15CH40
420						
430						
440						
450						
460		3.20	3.35			3.67
470	3.95			4.15	3.88	
480				4.46		
490						
500						
	Average (ns)					
	3.95	3.20	3.35	4.31	3.88	3.67

Table S5 Fluorescence lifetime of laurdan in lc-like phase at 37°C.

Wavelength (nm)	Fluorescence lifetime of laurdan in lc-like phase, 37°C (ns)					
	DPPC	CH10	CH40	SM15	SM15CH10	SM15CH40
420						
430						
440						
450						
460						
470		4.94*	5.84			5.93
480	5.59	5.92	6.2		5.59	6.10
490	5.86	5.94	6.19	5.59	5.57	6.10
500	5.82	5.89	6.15	5.75	5.56	6.11
	Average (ns)					
	5.76	5.92	6.10	5.67	5.57	6.06

*4.94 ns of CH10 at 470 nm was excluded from the calculation of the average lifetime as it is likely affected by the boundary phase, which was detected at 460 nm.

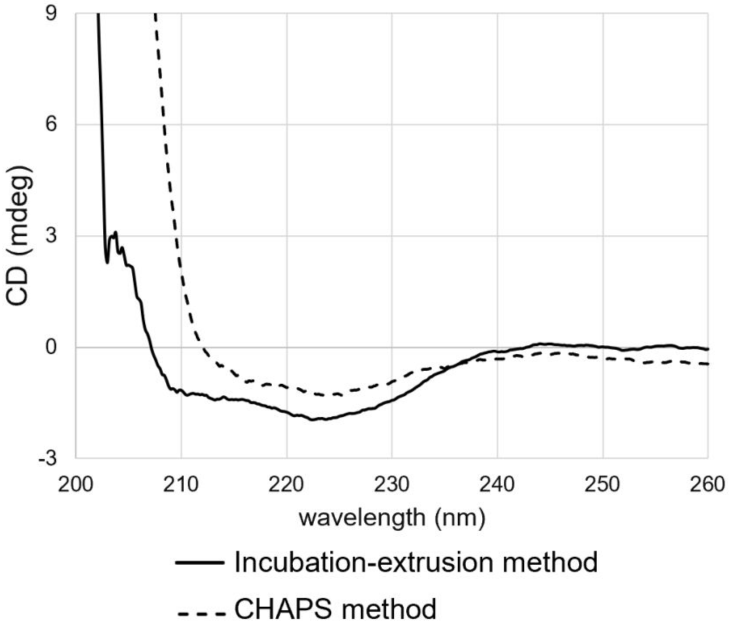

Figure S4 Comparison of CD spectra obtained by the incubation-extrusion method (solid line) and by incubation with 1% CHAPS followed by buffer exchange (dasshed line). α-helical CD spectrum was not observed with the latter condition.

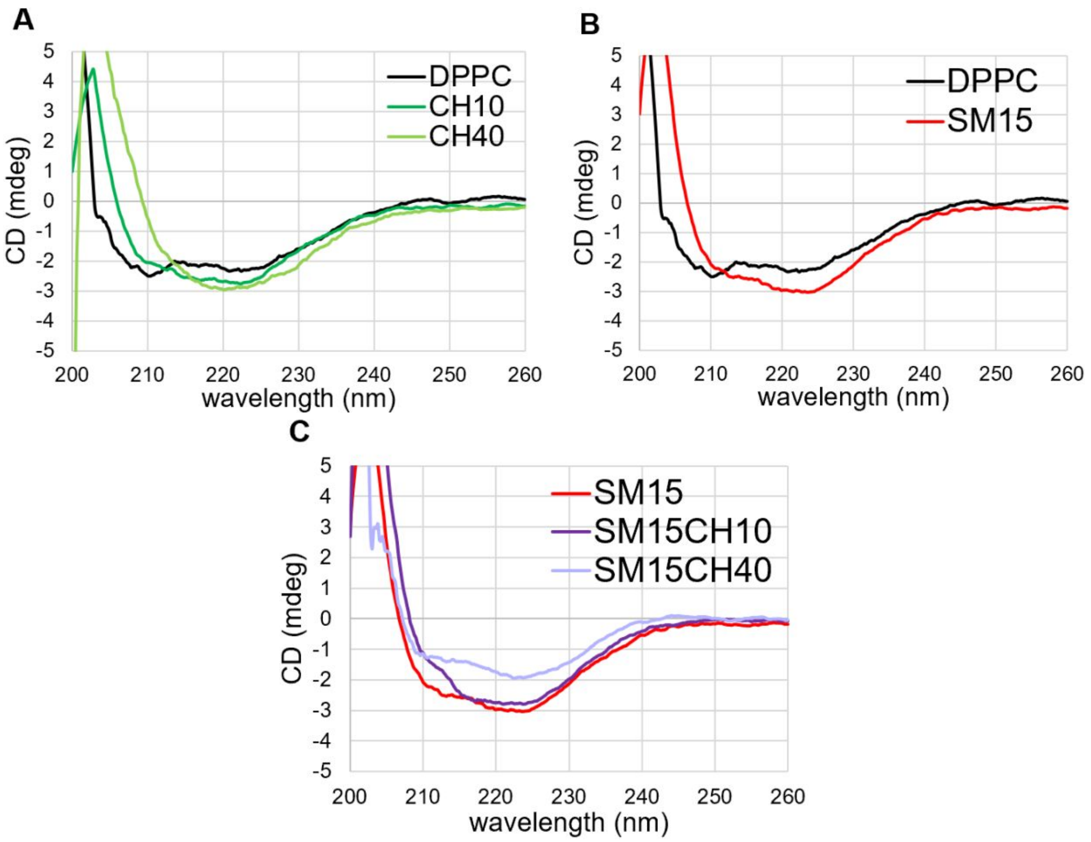

Figure S5 Impact of the lipid composition on CD spectra of the peptide; (A) CH addition to DPPC membrane, (B) SM addition to DPPC membrane, and (C) CH addition to DPPC-SM membrane.
